# The Comparability of Men Who Have Sex With Men Recruited From Venue-Time-Space Sampling and Facebook: A Cohort Study

**DOI:** 10.2196/resprot.3342

**Published:** 2014-07-17

**Authors:** Alfonso C Hernandez-Romieu, Patrick S Sullivan, Travis H Sanchez, Colleen F Kelley, John L Peterson, Carlos del Rio, Laura F Salazar, Paula M Frew, Eli S Rosenberg

**Affiliations:** ^1^Rollins School of Public HealthDepartment of EpidemiologyEmory UniversityAtlanta, GAUnited States; ^2^Division of Infectious DiseasesSchool of MedicineEmory UniversityAtlanta, GAUnited States; ^3^College of Arts and SciencesDepartment of PsychologyGeorgia State UniversityAtlanta, GAUnited States; ^4^Rollins School of Public HealthHubert Department of Global HealthEmory UniversityAtlanta, GAUnited States; ^5^School of Public HealthGeorgia State UniversityAtlanta, GAUnited States; ^6^Rollins School of Public HealthDepartment of Behavioral Sciences and Health EducationEmory UniversityAtlanta, GAUnited States

**Keywords:** men who have sex with men, MSM, Facebook, venue-based time sampling, online MSM, social media recruitment of MSM

## Abstract

**Background:**

Recruiting valid samples of men who have sex with men (MSM) is a key component of the US human immunodeficiency virus (HIV) surveillance and of research studies seeking to improve HIV prevention for MSM. Social media, such as Facebook, may present an opportunity to reach broad samples of MSM, but the extent to which those samples are comparable with men recruited from venue-based, time-space sampling (VBTS) is unknown.

**Objective:**

The objective of this study was to assess the comparability of MSM recruited via VBTS and Facebook.

**Methods:**

HIV-negative and HIV-positive black and white MSM were recruited from June 2010 to December 2012 using VBTS and Facebook in Atlanta, GA. We compared the self-reported venue attendance, demographic characteristics, sexual and risk behaviors, history of HIV-testing, and HIV and sexually transmitted infection (STI) prevalence between Facebook- and VTBS-recruited MSM overall and by race. Multivariate logistic and negative binomial models estimated age/race adjusted ratios. The Kaplan-Meier method was used to assess 24-month retention.

**Results:**

We recruited 803 MSM, of whom 110 (34/110, 30.9% black MSM, 76/110, 69.1% white MSM) were recruited via Facebook and 693 (420/693, 60.6% black MSM, 273/693, 39.4% white MSM) were recruited through VTBS. Facebook recruits had high rates of venue attendance in the previous month (26/34, 77% among black and 71/76, 93% among white MSM; between-race *P*=.01). MSM recruited on Facebook were generally older, with significant age differences among black MSM (*P*=.02), but not white MSM (*P*=.14). In adjusted multivariate models, VBTS-recruited MSM had fewer total partners (risk ratio [RR]=0.78, 95% CI 0.64-0.95; *P*=.01) and unprotected anal intercourse (UAI) partners (RR=0.54, 95% CI 0.40-0.72; *P*<.001) in the previous 12 months. No significant differences were observed in HIV testing or HIV/STI prevalence. Retention to the 24-month visit varied from 81% for black and 70% for white MSM recruited via Facebook, to 77% for black and 78% for white MSM recruited at venues. There was no statistically significant differences in retention between the four groups (log-rank *P*=.64).

**Conclusions:**

VBTS and Facebook recruitment methods yielded similar samples of MSM in terms of HIV-testing patterns, and prevalence of HIV/STI, with no differences in study retention. Most Facebook-recruited men also attended venues where VTBS recruitment was conducted. Surveillance and research studies may recruit via Facebook with little evidence of bias, relative to VBTS.

## Introduction

In the United States, men who have sex with men (MSM) are disproportionately affected by human immunodeficiency virus infection/acquired immunodeficiency syndrome (HIV/AIDS). At the end of 2009, MSM represented 52% of all people in the United States living with HIV, and are the only population for which new infections continue to increase [1]. Increases in HIV incidence are especially high among young MSM and MSM of color [2]. Recruiting valid samples of MSM is a key component of US HIV behavioral and clinical surveillance, and of research studies that seek to improve HIV prevention for MSM.

The National HIV Behavioral Surveillance System (NHBS) [3] and research studies [4-7] have relied, in part, on venue-based, time-space sampling (VBTS) [8] for the recruitment of MSM populations. In VBTS, after a period of formative research, venues (eg, bars, dance-clubs) attended by MSM are identified by health department staff members and calendars marking the days and times of venue attendance are created. Afterwards, venues, days, and times are randomly chosen for recruitment each month [8]. Although it is an effective method to recruit MSM and to minimize some types of biases, it has limitations. First, hiring, training, and retaining outreach staff is difficult as recruitment at venues requires staff that (1) are able to work highly variable schedules, including late nights and weekends, in outdoor settings, and poor weather, (2) collect valid and reliable data in many different venues, (3) minimize adverse events in venues, such as encounters with intoxicated and disorderly patrons, and (4) be culturally competent and racially diverse. Second, venues must be relatively safe for staff, a minimum of 2 staff members must be present during recruitment, and venue-owner approval of date/time block selected for recruitment is required, making VBTS logistically challenging. Third, support from the MSM community (eg, members of HIV prevention community planning groups, MSM community-based organizations, and advocacy groups) is crucial to formative research and the identification and access to important venues. Finally, seasonal patterns of venue attendance are an important barrier to recruitment [8]. Additionally, there are still concerns about how to best control for selection bias in VBTS due to the unequal sampling probabilities of participants, because MSM who visit gay venues more frequently are more likely to be sampled [8-11]. The limitations of VBTS, have led to an interest in developing other sampling methods that may allow researchers to complement VBTS by reaching comparable populations; the Internet and social media may present such an opportunity.

The growth of the Internet, social networking websites, and mobile technology usage, especially among minorities, have presented new opportunities for recruiting and studying MSM populations. In 2013, approximately 85% of adults living in the United States used the Internet [12], 71% of men aged 18 years of age or older were Facebook users, and 84% of all Internet users aged 18 to 29 had a Facebook account [13]. In a 2012 Pew survey, social media usage was higher among Hispanics (72%) and non-Hispanic blacks (68%) than among non-Hispanic whites (65%) [13]. Although there are few accurate estimates of Internet and social media usage by MSM, a meta-analysis by Liau et al [14] showed that 40% of MSM sought partners online. A more recent study among MSM social media users reported that 67% of study participants used social media to meet partners [15].

Online recruitment presents potential advantages over VBTS: it has a greater reach, is less time consuming, and less expensive [16,17]. However, online recruitment methods are not without limitations; Internet research has relied on convenience samples, and determining the source population limits the external validity of results [18]. A recent study identified that banner-advertisement recruitment methods may underrepresent minority MSM, MSM with less education, and MSM who do not identify as gay [19]. Finally, excluding duplicate responders and computer robots, which can perform a wide variety of automated tasks on the Internet, determining adequate compensation, ensuring confidentiality, and developing appropriate consent procedures continue to be important concerns [20].

The comparability of online and VBTS samples of MSM has recently received scrutiny, with the former method generally considered to yield higher risk samples [14,21-24]. To date, most Web-based studies of MSM have been cross-sectional with sampling occurring at specific online venues that are believed to contain higher-risk individuals (eg, Craigslist, Manhunt, Grindr), have had low minority representation, and used self-reported data on HIV or sexually transmitted infections (STI) [24-27]. Facebook is a social networking site where a wide range of social interactions occur and, in comparison with other online venues, is not specifically used for seeking sex partners. No studies to date have addressed the comparability of MSM recruited via VBTS and Facebook regarding venue attendance patterns, sexual and risk behaviors, and biological data about HIV and STIs. Finally, although the ability to recruit and retain a Web-based follow-up study using email and text messaging has been demonstrated [28,29], the relative success of retention in MSM in in-person studies recruited using online and VBTS methods is untested.

We performed a secondary analysis of Atlanta MSM recruited through VBTS and Facebook who enrolled in a longitudinal HIV prevention study with the objective of determining the comparability of samples of MSM recruited through VBTS and Facebook. To meet this objective we compared the recent venue and online site attendance, baseline behaviors, prevalent HIV/STI, and study retention by recruitment method. This analysis addressed a number of gaps; first, Web-based recruitment of MSM for an “in person” study has not been reported from Atlanta [17,25,30]; second, we enrolled a balanced sample of black and white MSM; third, we obtained laboratory-confirmed outcomes for HIV and STIs; and finally, the longitudinal nature of our study allowed us to quantify retention by mode of recruitment.

## Methods

### Recruitment and Enrollment

InvolveMENt (MEN is capitalized to highlight MSM) was a prospective cohort at Emory University designed to study the individual, dyadic, and community level factors that may contribute to the disparities in HIV and STI prevalence and incidence between black and white MSM in Atlanta, Georgia. The study methods have been described [31]. Briefly, approximately equal numbers of HIV-negative and HIV-positive black and white MSM were recruited from June 2010 to December 2012 using VBTS. Recognizing the growing importance of Facebook as a social space for MSM and to increase recruitment, Facebook was included as a venue within our sampling frame 6 months after enrollment began. Facebook encompasses a broad range of social interactions that aligned with the variety of venues sampled through VBTS (eg, bars, clubs, coffee shops, restaurants). Recruitment through Facebook took place from January 2011 to December 2012 via placement of banner advertisements.

The VBTS approach used was based on NHBS [8,32], and the initial venue sample frame was adopted from that used in Atlanta for the 2008 MSM cycle of the NHBS. Types of venues included in the sampling frame included bars, dance clubs, fitness clubs or gymnasiums, Gay Pride events, parks, restaurants, retail businesses, sex establishments, social organizations, street locations, and other special events. Venue-date-time units were randomly sampled. At sampled venue-day-time units, male attendees were systematically sampled and approached by the study staff and administered a recruitment script and screening questions using a hand-held device. For Facebook sampling, paid banner advertisements were placed in the Facebook advertising interface. Advertisements were delivered only to men 18 years of age or older who selected residing in Atlanta and interest in relationships with other men as demographic options on their Facebook profiles. Participants clicking on the banner advertisements were redirected to a Web-based survey where, after giving consent to screening, they were administered the same screening questions used for VBTS recruitment.

At the enrollment visit, potential participants were screened again for eligibility. Following the written informed consent process, participants were tested for HIV, chlamydia and gonorrhea (urethral and rectal), syphilis, and substances of abuse (urine dipstick) [31]. Participants completed a baseline computer-administered self-interview questionnaire. Those who tested HIV-negative were prospectively followed for 2 years, with visits at 3, 6, 9, 12, 18, and 24 months. This study was approved by the Emory University institutional review board (protocol 42405).

### Questionnaire Measures

The questionnaire used in InvolveMENt is published [31]; responses to the baseline questionnaire were used in the present analysis. To assess recent social media usage and Atlanta venue attendance, participants were asked the following: (1) Which of the following websites have you visited in the last month? (select all that apply): MySpace, Craigslist, Manhunt, FindFred, Black Gay Chat/BCGLive.com, Facebook, Adam4Adam, D-list, Friendster and OKCupid, (2) In the last month, in Atlanta, have you visited?: a) Bars/restaurants such as (8 local MSM-frequented bars/restaurants); b) Gyms such as (4 local MSM-frequented gyms); c) Clubs such as (3 local MSM-frequented clubs); d) Social gatherings such as (3 local MSM social groups); e) Outdoor locations such as (2 local MSM-frequented outdoor locations); or f) Bath houses such as (3 local MSM-frequented bath houses).

Sexual risk behaviors were collected as partner totals. Alcohol abuse was assessed using the CAGE scale (CAGE is an acronym of the 4 questions used in the scale [“Cut,” “Annoyed.” “Guilty,” “Eye-opener”]), a validated method used to measure alcohol dependence [33]. Using partnership-level responses on up to 5 most recent sex partners in 6 months, we created additional individual-level measures of partners met online, any serodiscussion [34], any sex with a discordant (ie, when one partner is infected but not the other) or unknown status partner, and alcohol or drug use at last sex.

### Analytical Methods

InvolveMENt used race-stratified sampling to ensure that equal numbers of black and white MSM were recruited, thus, to adhere to the design of our study and to understand race-specific aspects of recruitment all results were examined stratified by race. Participant use of social media and visits to Atlanta venues in the previous month was summarized and compared by recruitment method (VBTS and Facebook) using the Pearson chi-square test. For individuals recruited via VBTS, differences in Facebook use overall and by venue of recruitment were described.

Demographic characteristics were described for both recruitment methods overall and by race; differences were compared using Pearson chi-square tests. The age eligibility of participants was capped at 40 before Facebook recruitment began; participants 40 or older recruited through VBTS were excluded from the analysis (n=25). Partner counts were described as medians and group differences were tested using Wald chi-square tests in bivariate negative-binomial regression. Substance use, partnership attributes, HIV testing history, and prevalence of HIV/STI were summarized as proportions and tested using Pearson chi-square tests. The prevalence of urethral STI in our sample was too small to permit analysis. In comparisons where an expected cell count had <5 observations, Fisher’s exact test was used. To assess whether the within-race differences by recruitment methods differed among black and white MSM interactions of age, education, and partner counts with race were tested using likelihood ratio (LR) tests in logistic and negative binomial regression, respectively, and the Breslow-Day test was used to test interactions between race and employment, poverty, substance use, partnership attributes, HIV-testing history, and HIV/STI prevalence.

Due to significant differences in the age distribution between recruitment methods, we used multivariate models to assess whether levels of key outcomes differed when controlling for race and age. We used multiple multivariate logistic regression models to estimate the effect of recruitment for dichotomous outcomes, with comparisons made using predicted marginal prevalence ratios (PR) and Wald chi-square tests. For partner counts, outcomes were modeled using negative binomial regression, although Poisson and log-linear models were also considered. All models yielded similar parameter estimates and *P* values, and a goodness of fit test that compared the negative binomial and Poisson models [35] found that the negative binomial provided the best fit. Measures of association estimated in negative binomial models were risk ratios (RRs). All logistic and negative binomial models included race, age, and recruitment-method terms. Due to a borderline nonsignificant (*P*=.056) difference in HIV-positive proportions between Facebook and Atlanta-venue recruits among white MSM, baseline-HIV status was included in models of partner counts. An interaction term between recruitment method and race was included when modeling the outcome of syphilis infection because we detected a significant interaction using the Breslow-Day test, and was tested with a likelihood ratio test during our modeling procedure.

Retention to the 24-month visit was analyzed using the Kaplan-Meier method. Log-rank tests were performed to compare recruitment groups by race. All associations were considered significant at the α=.05 level. All analyses were performed using SAS V9.3.

## Results

### Recruitment and Costs

Screening for Involvement occurred from July 2010 through December 2012 with sampling occurring at 605 events located at 94 individual venues. Of 19,931 men approached at Atlanta venues, 45.07% (8,983/19,931) were screened, and 10.76% (2,144/19,931) were eligible on initial screening. Of 6,092 men who clicked on the Facebook advertisement, 22.32% (1,360/6,092) were screened, and 3.02% (184/6,092) were eligible on initial screening. The Facebook to VBTS cost per enrollee ratio was 0.75; the estimated costs of recruitment are available in Multimedia Appendix 1.

### Sample Characteristics and Venue Attendance

A total of 803 MSM were included in the analysis, of whom 13.7% (110/803) were recruited via Facebook and 86.3% (693/803) were recruited at Atlanta venues. Of Facebook recruits, 30.9% (34/110) were black MSM and 69.1% (76/110) were white MSM; overall 7.5% (34/453) of black MSM and 21.8% (76/349) of white MSM were recruited via Facebook. Among MSM recruited at Atlanta venues, 60.6% (420/693) were black and 39.4% (273/693) were white. Internet use and Atlanta venue attendance in the previous month are shown in Table 1. Among MSM recruited at Atlanta venues, 61.9% (260/420) of black and 58.2% (159/273) of white MSM had used Facebook in the previous month (between-race *P*=.31), and Facebook recruits were more likely to have used other forms of social media (eg, Craigslist, Manhunt, Adam4Adam) compared with Atlanta venue recruits. Among MSM recruited through Facebook, 77% (26/34) black MSM and 93% (68/76) white MSM had visited an Atlanta venue in the previous month (between-race *P*=.01). Facebook use differed by venue recruitment site ranging from 41% (24/58) for those recruited at street locations to 79% (38/48) for those recruited at cafes and restaurants (*P*<.001; data not shown).

### Demographic Characteristics, HIV Risk and Prevention Behaviors, and HIV/STI Prevalence

Demographic comparisons by recruitment method are shown in Table 2. MSM recruited on Facebook were generally older, with significant age differences among black MSM (*P*=.02), but not white MSM. White MSM recruited at Atlanta venues had a higher education level than their Facebook counterparts (*P*=.01), with no difference among black MSM. Conversely, MSM recruited at Atlanta venues were more likely to report current employment compared with Facebook recruits (*P=*.047), with significant differences among black MSM (*P=*.04) but not white MSM (*P*=.05). Interactions between race and demographic variables were not significant.

The number of total and UAI partners in the previous 12 months were higher for Facebook recruits compared with Atlanta-venue recruits overall and among white MSM (Table 3). Facebook recruits were more likely to report having met at least 1 partner online (*P=*.006). Tests of interaction between race and recruitment type for risk behavior outcomes, substance use, partnership attributes, and HIV-testing were all non-significant.

**Table 1:** Internet use and real-world venue attendance in the previous month by recruitment type (Facebook vs Atlanta Venues) and race, InvolveMENt, Atlanta, GA.

		Total			Black				White		
		Facebook	Atlanta venue	*P*	Facebook	Atlanta venue	*P*		Facebook	Atlanta venue	*P*
		(n=110)	(n=693)		(n=34)	(n=420)			(n=76)	(n=273)	
		n (%)			n (%)				n (%)		
**Internet use **											
	Used Facebook	110/110 (100.0)	419/692 (60.5)	-	34/34 (100.0)	260/419 (62.1)	-		76/76 (100.0)	159/273 (58.2)	-
	Visited at least one Internet site in the past month^a^	96/110 (87.3)	332/693 (47.9)	<.001	28/34 (82.4)	216/420 (51.4)	<.001		68/76 (89.5)	116/273 (42.5)	<.001
**Real-world**											
	Attended at least 1 venue in the past month^b^	97/110 (88.2)	436/448 (97.3)	<.001	26/34 (76.5)	273/283 (96.5)	<.001		71/76 (93.4)	163/165 (98.8)	.03

^a^Does not include Facebook.

^b^Total offline venue attendance does not add up to 100% because venue-based, time-space sampling (VBTS) also sampled at street locations and during Gay Pride, thus all VBTS recruits did not necessarily visit one of the venues in the previous month.

**Table 2:** Demographic characteristics by recruitment type (Facebook vs Atlanta Venues) and race InvolveMENt, Atlanta, GA.

Demographics		Total			Black			White			Race Interaction
		Facebook	Atlanta venue	*P*	Facebook	Atlanta venue	*P*	Facebook	Atlanta venue	*P*	*P*
		(n=110)	(n=693)		(n=34)	(n=420)		(n=76)	(n=273)		
		n (%)			n (%)			n (%)			
**Age category**		n=110	n=693	.03	n=34	n=420	.02	n=76	n=273	.14	.69
	18-19	11/110 (10.0)	32/668 (4.8)		5/34 (14.7)	22/411 (5.4)		6/76 (7.9)	10/257 (3.9)		
	20-24	24/110 (21.8)	223/668 (33.4)		5/34 (14.7)	51/411 (36.7)		19/76 (25.0)	72/257 (28.0)		
	25-29	35/110 (31.8)	204/668 (30.5)		14/34 (41.2)	123/411 (29.9)		24/76 (31.6)	81/257 (31.5)		
	30-39	34/110 (30.9)	209/668 (31.3)		10/34 (29.4)	115/411 (28.0)		27/76 (35.5)	94/257 (36.6)		
	40+^a^	N/A	n=25		N/A	n=9		N/A	n=16		
**Education**		n=110	n=689	.10	n=34	n=417	.11	n=76	n=272	.01	.45
	<High school	2/110 (1.8)	16/689 (2.3)		2/34 (6.0)	14/417 (3.4)		0/76 (0.0)	2/272 (0.7)		
	High school /general educated development	22/110 (20.0)	111/689 (16.1)		10/34 (29.4)	89/417 (21.3)		12/76 (15.8)	22/272 (8.1)		
	Some college	53/110 (48.2 )	272/689 (39.5)		18/34 (52.9)	183/417 (43.9)		35/76 (46.1)	89/272 (32.7)		
	College or >	33/110 (30.0)	290/689 (42.1)		4/34 (11.8)	131/417 (31.4)		29/76 (38.2)	159/272 (58.5)		
Employed		72/107 (67.3)	526/690 (76.2)	.047	17/31 (54.8)	301/417 (72.2)	.04	55/76 (72.4)	225/273 (82.4)	.05	.71
Poverty		19/95 (20.0)	119/593 (20.1)	.99	9/25 (36.0)	88/342 (25.7)	.26	10/70 (14.3)	31/251 (12.4)	.67	.59

^a^Enrollment through Facebook took place after the maximum recruitment age was capped at 40, thus no percentages are presented for this category.

**Table 3:** HIV risk and prevention behaviors by recruitment type (Facebook vs Atlanta Venues) and race, InvolveMENt, Atlanta, GA.

		Total			Black			White			Race Interaction
		Facebook	Atlanta venue	*P*	Facebook	Atlanta venue	*P*	Facebook	Atlanta venue	*P*	*P*
		(n=110)	(n=693)		(n=34)	(n=420)		(n=76)	(n=273)		
		Median (IQR)			Median (IQR)			Median (IQR)			
**Risk behaviors** ^**a,b**^											
	Total # male partners	8 (4-15)	6 (3-10)	.002	5 (3-9)	5 (3-10)	.92	9 (5-20)	7 (4-12)	.005	.45
	Main male partners	1 (0-2)	1 (0-2)	.98	1 (0 - 2)	1 (1-2)	.34	1 (0-2)	1 (0-2)	.06	.45
	Casual male partners	5.5 (3-12)	4 (2-10)	.03	4 (2 - 8)	4 (2-8)	.88	7 (3-18)	6 (3-11)	.06	.23
	UAI partners	2 (1-5)	1 (0-3)	<.001	2 (1 – 3.5)	1 (0-3)	.16	2 (1-5)	1 (1-3)	<.001	.17
**Substance use, n (%)**											
	Alcohol dependence	21/110 (19.1)	204/693 (29.3)	.03	4/34 (11.8)	93/420 (22.1)	.16	17/76 (22.3)	111/273 (40.7)	.003	.86
	Marijuana^c^	26/110 (23.6)	162/693 (23.4)	.95	9/34 (26.5)	112/420 (26.7)	.98	17/76 (22.3)	50/273 (18.3)	.43	.61
	Cocaine^c^	4/110 (3.6)	51/693 (7.4)	.15	2/34 (5.9)	34/420 (8.1)	1	2/76 (2.6)	17/273 (6.3)	.39	.6
	Other noninjection drugs^c^	8/110 (7.3)	14/693 (2.0)	.006	2/34 (5.9)	8/420 (1.9)	.17	6/76 (7.9)	6/273 (2.2)	.03	.87
**Partnership attributes, n (%)**											
	Met at least 1 partner online	79/110 (71.8)	402/693 (58.0)	.006	22/34 (64.7)	234/420 (55.7)	.31	57/76 (75.0)	168/273 (61.5)	.03	.59
	Discussed serostatus with at least 1 partner	94/110 (85.5)	551/693 (79.5)	.14	26/34 (76.5)	310/420 (73.8)	.73	241/273 (88.3)	68/76 (89.5)	.77	.97
	Any discordant partner	78/110 (70.9)	482/693 (69.6)	.77	26/34 (76.5)	314/420 (74.8)	.83	52/76 (68.4)	168/273 (61.5)	.27	.68
	Used alcohol or drugs at last sex	33/110 (30.0)	209/693 (30.2)	.95	110/420 (26.2)	9/34 (26.5)	.97	24/76 (31.6)	96/273 (35.2)	.56	.72
**HIV-testing history, n (%)**											
	Lifetime	100/110 (90.9)	648/691 (93.7)	.26	31/34 (91.2)	386/418 (92.3)	.74	69/76 (90.8)	262/331 (79.2)	.07	.82
	Lifetime^d^	74/84 (88.1)	508/551 (92.2)	.21	20/23 (87.0)	269/301 (89.4)	.73	54/61 (88.5)	239/250 (95.6)	.06	.32
	Previous 12 months^d^	59/84 (70.2)	384/550 (69.8)	.94	17/23 (73.9)	197/300 (66.7)	.42	42/61 (68.9)	187/250 (74.8)	.35	.23

^a^Partners in the previous 12 months.

^b^Extreme values capped at the following values: a) 125 total partners in the previous 12 months; b) 25 main male partners in the previous 12 months; c) 100 casual partners in the previous 12 months; and d) 125 UAI partners in the previous 12 months.

^c^Urine-detected.

^d^Excluding HIV+ aware.

HIV and STI prevalence at baseline is presented in Table 4. HIV test results at baseline were similar for MSM recruited via Facebook and Atlanta venues within each racial group. Although within-race differences between Facebook and Atlanta-venue recruits were nonsignificant, Atlanta-venue recruits overall were more likely to be infected with a rectal STI compared with Facebook recruits. The significant interaction between race and recruitment method *(P*=.03) for syphilis indicates that while within-race differences between Atlanta-venue and Facebook recruits were not significant, there were significant differences between black and white MSM recruited via Facebook as well as black and white MSM recruited at Atlanta venues.

Multivariate models adjusting simultaneously for race and age are presented in Table 5, and were similar to results obtained in bivariate analyses. Atlanta-venue recruits had fewer total partners (RR=0.79, 95% CI 0.65-0.95) and UAI partners (RR=0.56, 95% CI 0.42-0.73) in the previous 12 months compared with Facebook recruits. No differences were observed in testing patterns or prevalence of infection in multivariate models, and the prevalence of HIV/STI were similar by recruitment method.

**Table 4:** HIV and STI prevalence at baseline by recruitment type (Facebook vs Atlanta Venues) and race, InvolveMENt, Atlanta, GA.

Infections	Total			Black			White			Race Interaction
	Facebook	Atlanta venue	*P*	Facebook	Atlanta venue	*P*	Facebook	Atlanta venue	*P*	*P*
	(n=110)	(n=693)		(n=34)	(n=420)		(n=76)	(n=273)		
	n (%)			n (%)			n (%)			
HIV (+)	28/110 (25.5)	215/693 (31.0)	.24	13/34 (38.2)	184/420 (43.8)	.53	15/76 (19.7)	31/273 (11.4 )	.06	.08
Rectal STI	2/110 (1.8)	51/693 (7.4)	.03	1/34 (2.9)	45/420 (10.7)	.23	1/76 (1.3)	6/273 (2.2)	1	.56
Syphilis	11/110 (10.0)	112/690 (16.2)	.09	4/34 (11.8)	99/418 (23.7)	.13	7/76 (9.2)	13/273 (4.8)	.16	.03

**Table 5:** Adjusted multivariate models for risk behaviors, testing, and infections at baseline by recruitment type (Facebook vs Atlanta Venues) adjusting for age and race, InvolveMENt, Atlanta, GA.

Characteristics		Ratio measures	*P*
**Risk behaviors** ^a^ **(ref = Facebook), RR (95% CI)** ^d^			
	Total # male partners	0.79 (0.65-0.95)	.01
	Main male partners	0.93 (0.74-1.16)	.46
	Casual male partners	0.81 (0.65-1.01)	.06
	UAI partners	0.56 (0.42-0.73)	<.001
**HIV-testing history (ref = Facebook), PR (95% CI)** ^e^			
	Lifetime	1.03 (0.96-1.11)	.26
	Lifetime^b^	1.05 (0.96-1.15)	.18
	Previous 12 months^b^	1.01 (0.86-1.18)	.91
**HIV infection and rectal STI** ^c^ **(ref = Facebook), PR (95% CI)** ^e^ ** **			
	HIV Infection	0.84 (0.63-1.12)	.23
	Rectal STI	1.91 (0.58-6.22)	.21
	Syphilis (white MSM)	0.50 (0.21-1.22)	.15
	Syphilis (black MSM)	1.92 (0.76-4.85)	.13

^a^Partners in the previous 12 months.

^b^Excludes participants who were aware of their HIV-positive status.

^c^The prevalence of urethral STI in the cohort was too small to permit analysis.

^d^Models adjusted for race, age, and HIV-status at baseline.

^e^Models adjusted for race and age.

### Retention

**Figure 1:**
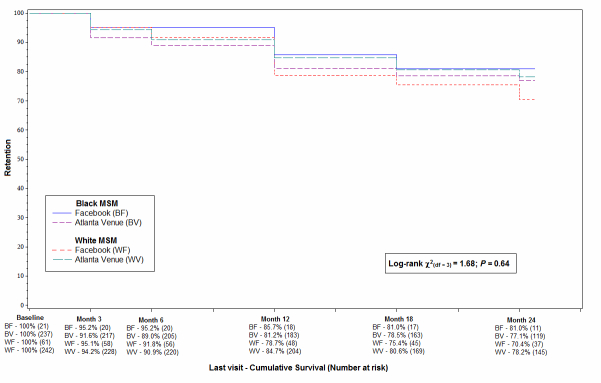
Study retention by recruitment type (Facebook vs Atlanta Venues) and race, InvolveMENt, Atlanta, GA.

Study retention by race for Facebook and Venue recruits is shown in Figure 1. A total of 561 individuals were followed prospectively of whom 44.2% (248/561) were black MSM. Race-recruitment method-specific retention to the 24-month visit estimates were 81.0% for black and 70.4% for white MSM recruited via Facebook, and 77.1% for black and 78.2% for white MSM recruited at venues. There was no statistically significant difference in retention among the four groups (log-rank *P*=.64).

## Discussion

### Principal Findings

Comparing Facebook and VBTS samples of MSM, we observed substantial real-world venue attendance and comparable levels of HIV-testing history and prevalent HIV/STI, with some differences in risk behaviors. We found no difference in 24-month retention rates between recruitment methods. Additionally, cost per screening completed and per study participant were lower for Facebook relative to VBTS. Our findings indicate that recruiting through Facebook may yield comparable samples with those obtained by current VBTS recruitment with similar retention and at a lower cost.

Most Facebook users in our sample visited at least one Atlanta venue where VTBS recruitment might take place, with lower venue attendance rates among black MSM. This corroborates previous findings by Sanchez et al [23], who reported that 95% of MSM recruited online had visited a venue in the last year, with lower rates among minority MSM. Although these findings support the notion that MSM recruited via Facebook might also have been sampled from Atlanta venues, it is unclear whether Facebook recruits would have participated in the study had they been approached at Atlanta venues. More research is needed to explore whether significant differences exist between MSM recruited via Internet and VBTS in factors motivating them to take part in research studies. Additionally, while 88.2% (97/110) of our Facebook sample had visited a real-world venue in the last month, 60.6% (419/692) of VBTS recruits indicated having used Facebook in the same time period. This difference may be due to the oversampling of certain venues, because Facebook use differed by venue of recruitment; the difference might also be driven by the higher number of white MSM recruited through Facebook, because they reported lower proportions of Facebook use in the previous month. Nevertheless, previous research has shown that there are significant differences in samples obtained at different venues [36], the expansion of this research to differences in the use of social media among different samples of MSM may provide insight into ways of complementing VBTS samples.

Demographic comparisons among white MSM yielded similar findings to other studies comparing online and offline recruitment, in which online recruits were reported to have a higher level of formal education [17,23]. Age differences by recruitment method were only observed among black MSM, with younger black MSM more likely to be recruited via Facebook. Sampling young black MSM is of interest due to the increased number of infections observed in this population [2]. Facebook and other social media may be useful ways to reach these MSM, because they are less likely to be recruited at real-world venues and may be under age for admittance to many gay venues. Regardless, we found that after adjusting for these differences in race and age, there were no systematic biases between VBTS and Facebook sampling in HIV or STI infections or HIV testing patterns. While more research is needed to understand the characteristics of MSM recruited through Facebook, studies seeking to recruit online samples of MSM with specific age or race subgroups may use quota/stratified methods as an alternative to adjust for demographic characteristics of Facebook users [37]. Additionally, certain strategies, such as race-matched banner ads have shown to increase the effectiveness of recruiting online samples of minority MSM [19].

Previous samples of MSM obtained in online chat-rooms and sex-seeking Internet spaces were found to be a higher risk population than MSM attained through VBTS [14,23,24]. In our study, Facebook recruits were more likely to report having visited MSM websites compared with Atlanta venue recruits, indicating that the former may engage in more online sex-seeking behaviors. However, while the higher number of total and UAI partners in the previous 12 months among Facebook supports the idea that they may be a higher risk group, based on comparisons of HIV/STI prevalence, our results indicate no difference in risk between Facebook and Atlanta venue recruits. Additionally, contrary to previous findings, we found that partner serodiscussion and serodiscordance were not significantly different between Web-based and venue samples [17,24,26]. Facebook encompasses a wider range of social interactions than the more sex-seeking focused online environments, as such, it may contain a wider range of risk samples. Therefore, researchers seeking to sample MSM with similar characteristics as those encountered at real-world venues should consider sampling through general social networking sites, while researchers wishing to do studies of higher-risk MSM should consider sampling through online sex-seeking sites.

Our study showed comparable retention rates among all four race-recruitment method groups at 24 months, with no statistically significant difference across groups. Furthermore, retention rates were relatively high relative to a recently reported cohort study [38]. InvolveMENt used a variety of retention-enhancing techniques such as using a custom-built database system that managed participant tracking, scheduling, and communications (including automatically sent visit reminders), as well as routine check-ins from study staff via cell phone, emails, and/or text messaging. Using appropriate and engaging retention methods is paramount regardless of the source of recruitment.

Finally, the average costs associated with recruiting through Facebook were moderately lower than those associated with recruitment via VBTS in terms of cost per completed screening and cost per enrollee in our study. This indicates that studies may potentially recruit MSM more efficiently via Facebook.

### Limitations

There are several limitations of this analysis. First, we limited Facebook recruitment to men indicating interest in other men on their profiles. As of April 2014, approximately 1.05% (11,600 out of 1.1 million) of men 18 years of age or older living within a 25 mile radius of Atlanta indicated interest in other men as their sexual orientation of Facebook; this is most likely an underestimate of the number of MSM using Facebook. Our results should be interpreted with caution as our sample is not representative of black and white MSM who do not disclose their sexual orientation in Facebook. Second, the demographic characteristics of MSM recruited through Facebook may change over time. Multiple and repeated samples of MSM using Facebook may be needed to quantify these changes and to ensure the comparability of VBTS and Facebook samples of MSM. Third, we were unable to compare Facebook participants starting but failing to complete the Web-based screening survey to those completing the survey. Fourth, the sample of black MSM recruited through Facebook was small and we therefore had low statistical power to detect differences for the two samples of black MSM and differences between races. Finally, because we used Facebook as a sampling venue and not a sampling frame separate from VBTS, we were unable to compare the effectiveness VBTS and Facebook in recruiting large samples of MSM.

### Conclusions

Online social networks such as Facebook may enable recruitment of samples of MSM that are similar to samples attained through VBTS. In our study, VBTS and Facebook recruitment methods yielded similar samples of MSM in terms of HIV-testing history and prevalence of HIV/STI, with no differences in study retention. Surveillance and research studies may recruit via Facebook with little evidence of bias, relative to VBTS, and potentially more efficiently. Furthermore, highly retained samples of black and white MSM may be recruited to traditional prospective studies via Facebook. These findings support the comparability of the two sampling methods, although it is unclear how either method compares with the broader, underlying source population of MSM. Future research should seek to characterize MSM populations that are missed by traditional recruitment methods such as VBTS.

## Multimedia Appendix 1

Approximate costs of VBTS and Facebook recruitment, InvolveMENt, Atlanta, GA.

## Abbreviations

CAGE Scale: CAGE is an acronym of the 4 questions used in the scale (“Cut,” “Annoyed.” “Guilty,” “Eye-opener”)
HIV/AIDS: human immunodeficiency virus/acquired immunodeficiency syndrome
LR: likelihood ratio
MSM: men who have sex with men
NHBS: national HIV behavioral surveillance system
PR: prevalence ratios
RR: risk ratio
STI: sexually transmitted infection
UAI: unprotected anal intercourse
VBTS: venue-based, time-space sampling
