# Supplementary material for: The Comparability of Men Who Have Sex With Men Recruited From Venue-Time-Space Sampling and Facebook: A Cohort Study
Source: JMIR Res Protoc. 2014 Jul 17;3(3):e37. doi: 10.2196/resprot.3342 (PMC4129125; doi:10.2196/resprot.3342)
Supplement: Supplementary file 1 [file resprot_v3i3e37_app1.pdf]

Appendix 1. Approximate costs of VBTS and Facebook recruitment, InvolveMENt, Atlanta, GA

**Recruitment characteristics**	**VBTS**		**Facebook**	
Total cost ($US)[a]	63,173		7,550	
	n	Cost($) / n	n	Cost($) / n
Total number of approaches/impressions	19,931	3.2	19,580,845	0.0004
Completed screenings	8,983	7.0	1,360	5.6
Final enrollees[b]	693	91.2	110	68.6

[a] Approximate cost: VBTS - includes salary of recruiters stationed at Atlanta venues, survey instruments, survey programs, and logistics coordinator salary, Facebook - includes advertisement costs only

[b] Constitute the final sample for analysis in our cohort
